# Circulating tumor cells criteria (CyCAR) versus standard RECIST criteria for treatment response assessment in metastatic colorectal cancer patients

**DOI:** 10.1186/s12967-018-1624-2

**Published:** 2018-09-06

**Authors:** Mayte Delgado-Ureña, Francisco G. Ortega, Diego de Miguel-Pérez, Alba Rodriguez-Martínez, Jose L. García-Puche, Hugh Ilyine, Jose A. Lorente, Jose Exposito-Hernandez, M. Carmen Garrido-Navas, Miguel Delgado-Ramirez, M. José Serrano

**Affiliations:** 1 Integral Oncology Division, Clinical University Hospital, Av. Dr. Olóriz 16, 18012 Granada, Spain; 2 Liquid Biopsy and Metastasis Research Group, GENYO, Centre for Genomics and Oncological Research, Pfizer/University of Granada/Andalusian Regional Government PTS, Granada, Avenida de la Ilustración, 114, 18016 Granada, Spain; 3 Laboratory of Genetic Identification, Legal Medicine and Toxicology Department, Faculty of Medicine, University of Granada, Avenida de la Investigación, 11, 18071 Granada, Spain; 4 DestiNA Genomics Ltd, 7-11 Melville St, Edinburgh, EH3 7PE UK; 5 Division of Preventive Medicine and Public Health, CIBERESP, University of Jaen, Campus de las Lagunillas, 23072 Jaén, Spain

**Keywords:** Metastatic colorectal cancer, Bevacizumab, Circulating tumor cells, RECIST, CyCAR, Prognosis

## Abstract

**Background:**

The use of circulating tumor cells (CTCs) as indicators of treatment response in metastatic colorectal cancer (mCRC) needs to be clarified. The objective of this study is to compare the Response Evaluation Criteria in Solid Tumors (RECIST) with the Cytologic Criteria Assessing Response (CyCAR), based on the presence and phenotypic characterization of CTCs, as indicators of FOLFOX–bevacizumab treatment response.

**Methods:**

77 mCRC blood samples from FOLFOX–bevacizumab treated patients were analyzed to isolate CTCs before and after (12 and 24 weeks) treatment, using an immunomagnetic separation method. VEGFR expression was identified by double immunostaining.

**Results:**

We observed a decrease of CTCs (42.8 vs. 18.2%) and VEGFR positivity (69.7% vs. 41.7%) after treatment. According to RECIST, 6.45% of the patients did not show any clinical benefit, whereas 93.55% patients showed a favorable response at 12 weeks. According to CyCAR, 29% had a non-favorable response and 71% patients did not. No significant differences were found between the response assessment by RECIST and CyCAR at 12 or 24 weeks. However, in the multivariate analysis, RECIST at 12 weeks and CyCAR at 24 weeks were independent prognostic factors for OS (HR: 0.1, 95% CI 0.02–0.58 and HR: 0.35, 95% CI 0.12–0.99 respectively).

**Conclusions:**

CyCAR results were comparable to RECIST in evaluating the response in mCRC and can be used as an alternative when the limitation of RECIST requires additional response analysis techniques.

## Background

In colorectal cancer (CRC), metastasis is the main cause of death [1]. Distant metastasis is identified in approximately 25% of patients at initial diagnosis, and half of CRC patients will develop it [2]. During this process, circulating tumor cells (CTCs), detach from primary sites, enter the bloodstream and extravasate in distant organs. CTCs are now being studied in order to have a deeper understanding of the metastatic processes [3]. The phenotypic and genetic characterization of CTCs is especially important; as different subpopulations of CTCs can be detected in the blood of these patients [4]. These CTCs subclones can depict in real time the heterogeneity of a tumor, displaying its different abilities to elude therapies, and therefore, determining tumor response to treatment [5].

Metastatic colorectal cancer patients (mCRC) are currently subjected to a treatment regime combining chemotherapy with biological therapies. Bevacizumab, a monoclonal antibody inhibits the tyrosine kinase vascular endothelial growth factor A (VEGF-A) and blocks its transduction signal, through both VEGFR-1 and VEGFR-2. VEGF-A is a potent pro-angiogenic growth factor that stimulates proliferation, migration, and survival of endothelial cells. As it is one of the more important proteins expressed by tumor cells, VEGF is an important target of anticancer therapies. Cancer cells and tissues with high metabolic rates are characterized by hypoxia, which induces the transcription of VEGF protein [6]. Circulating VEGF binds with high affinity to VEGF receptors (VEGFR-1 and VEGFR-2) and its co-receptors neuropilin (NRP-1 and NRP-2), which are expressed on the surface of endothelial cells and play a critical role in the development of angiogenesis, by stimulating recruitment and proliferation of endothelial cells [7]. Bevacizumab is an IgG1 recombinant humanized monoclonal antibody that acts by selectively binding to circulating VEGF-A, creating a large molecule that renders it unable to bind to its cell surface receptors, reducing microvascular growth of tumor blood vessels and limiting blood supply of nutrients and oxygen to tumor tissues. In combination with intravenous 5-fluorouracil-based chemotherapy, it is indicated for first- or second-line treatment of patients with metastatic colorectal cancer. In combination with fluoropyrimidine–irinotecan- or fluoropyrimidine–oxaliplatin-based chemotherapy, it is indicated for second-line treatment of patients with metastatic colorectal cancer who have progressed on a first-line Bevacizumab containing regimen [8, 9]. First line bevacizumab has been demonstrated to improve overall survival (OS), progression-free survival (PFS) and treatment response rate in mCRC [10]. Despite these improvements, most mCRC patients will die due to disease progression [11]. With this in mind, extensive biomarker programs have now been built into numerous clinical studies with bevacizumab. However, predictive markers for bevacizumab treatment have yet to be validated [12].

In this clinical experimental work, we aimed to establish the predictive role of CTCs, and their expression of a treatment-associated marker (VEGFR), as response biomarkers to bevacizumab in mCRC patients, as well as their relationship with disease progression and death risk. Furthermore, we then compared the Response Evaluation Criteria in Solid Tumors (RECIST) version 1.1 [13] with our proposed Cytologic Criteria Assessing Response (CyCAR), based on CTC status, to determine their respective utility as predictive and prognostic assessments. Finally, we compared treatment responses of mCRC patients under FOLFOX–bevacizumab-containing chemotherapy by both criteria.

## Methods

### Study design

We conducted a prospective longitudinal cohort study of 77 patients with mCRC who underwent first-line treatment with FOLFOX6 m (Oxaliplatin 85 mg/m^2^, Leucovorin 400 mg/m^2^, 5-FU 400 mg/m^2^ bolus and 5-FU 2400 mg/m^2^ over 46 h) and bevacizumab (5 mg/kg) every 2 weeks until disease progression, at the Department of Oncology, San Cecilio University Hospital in Granada (Spain), between April 2011 and November 2015. Control blood samples were drawn from 16 healthy volunteers with no history of malignant disease.

The study was conducted in accordance with the Declaration of Helsinki and approved by the ethical Committee of the Hospital. Written informed consent was obtained from every cancer patient and healthy volunteer.

None of the patients had received any other type of biological treatment before inclusion in the study. Computed tomography of the chest, abdomen and pelvis was performed at baseline, at 12 weeks, at 24 weeks and finally each 12 weeks until death. Image interpretation was performed using RECIST, to classify each disease as complete response, partial response, stable disease, or progressive disease. Patients who died before a follow-up imaging study were classified as having progressive disease. According to patient response to the therapy, they were divided into two groups: those with favorable response, including patients who have achieved complete response, partial response or stable disease, and those with non-favorable response, including patients without clinical benefit (progressive disease or death).

Median follow-up time for all patients was 23.3 months (range 2–105 months). Clinical outcomes were evaluated in terms of PFS and OS. PFS was defined as the elapsed time from the start of the treatment to progression or death. OS was defined as the elapsed time from the start of the treatment to death.

Data was collected for the following variables: age, gender, primary tumor location, metastasis surgery, primary tumor surgery, synchronous metastasis, K-RAS status, Basal CEA, Basal Ca 19.9, progression, survival, RECIST and CyCAR responses (Table 1).

**Table 1:** Clinicopathological characteristics of the patients according to the CyCAR criteria at baseline status (CTC1) and VEGFR status

	CTC 1			CTC 1 VEGFR		
	N (%) −	N (%) +	*p*	N (%) −	N (%) +	*p*
Age (years)						
< 55	15 (62.5%)	9 (37.5%)	0.622	3 (33.3%)	6 (66.7%)	1.00
≥ 55	29 (54.7%)	24 (45.3%)		7 (29.2%)	17 (70.8%)	
Gender						
Male	30 (58.2%)	21 (41.2%)	0.808	9 (40.9%)	13 (59.1%)	0.144
Female	14 (53.9%)	12 (46.1%)		2 (16.7%)	10 (83.3%)	
Primary tumor location						
Colon	25 (54.4%)	21 (45.6%)	0.641	5 (23.8%)	16 (76.2%)	0.164
Rectum	19 (61.3%)	12 (38.7%)		6 (46.2%)	7 (53.8%)	
Metastasis surgery						
No	32 (57.1%)	24 (42.9%)	1.00	8 (32%)	17 (68%)	0.625
Yes	12 (57.1%)	9 (42.9%)		3 (33.3%)	6 (66.7%)	
Primary tumor surgery						
No	21 (75%)	7 (25%)	0.019*	4 (57.1%)	3 (42.9%)	0.132
Yes	23 (46.9%)	26 (53.1%)		7 (25.9%)	20 (74.1%)	
Synchronous metastasis						
No	3 (25%)	9 (75%)	0.024*	2 (22.2%)	7 (77.8%)	0.686
Yes	41 (63.1%)	24 (36.9%)		8 (33.3%)	16 (66.7%)	
K-RAS						
Mutated	25 (69.4%)	11 (30.6%)	0.061	4 (36.4%)	7 (63.6%)	0.546
Wild-type	18 (46.2%)	21 (53.8%)		7 (31.8%)	22 (68.2%)	
Basal CEA						
Standard	6 (40%)	9 (60%)	0.153	2 (20%)	8 (80%)	0.339
High	37 (61.7%)	23 (38.3%)		8 (34.8%)	15 (65.2%)	
Basal Ca 19.9						
Standard	19 (46.3%)	22 (53.7%)	0.034*	7 (31.8%)	15 (68.2%)	0.627
High	23 (71.9%)	9 (28.1%)		3 (30%)	7 (70%)	
Progression						
Yes	28 (53.8%)	24 (46.2%)	0.485	7 (29.2%)	17 (70.8%)	0.634
No	9 (64.3%)	5 (35.7%)		2 (40%)	3 (60%)	
Survival						
Yes	18 (60%)	12 (40%)	1.00	3 (25%)	9 (75%)	0.683
No	22 (61.1%)	14 (38.9)		5 (35.7%)	9 (64.3%)	
RECIST 12 weeks						
Favorable	31 (53.4%)	27 (46.6%)	0.402	7 (25.9%)	20 (74.1%)	0.557
Adverse	3 (75%)	1 (25%)		0 (0%)	1 (100%)	
RECIST 24 weeks						
Favorable	19 (52.8%)	17 (47.2%)	0.376	4 (23.5%)	13 (76.5%)	0.468
Adverse	5 (38.5%)	8 (61.5%)		3 (37.5%)	5 (62.5%)	
CyCAR 12 weeks						
Favorable	29 (65.9%)	15 (34.1%)	0.087	4 (26.7%)	11 (73.3%)	1.00
Adverse	7 (38.9%)	11 (61.1%)		3 (27.3%)	8 (72.7%)	
CyCAR 24 weeks						
Favorable	30 (55.6%)	24 (44.4%)	0.537	7 (29.2%)	17 (70.8%)	0.574
Adverse	8 (66.7%)	4 (33.3%)		2 (50%)	2 (50%)	

*: Statistically significant

CyCAR: Cytologic Criteria Assessing Response; CTC: circulating tumor cell; VEGFR: vascular endothelial growth factor receptor; RECIST: Response Evaluation Criteria in Solid Tumors; *p*: *p* value

**Fig. 1:**
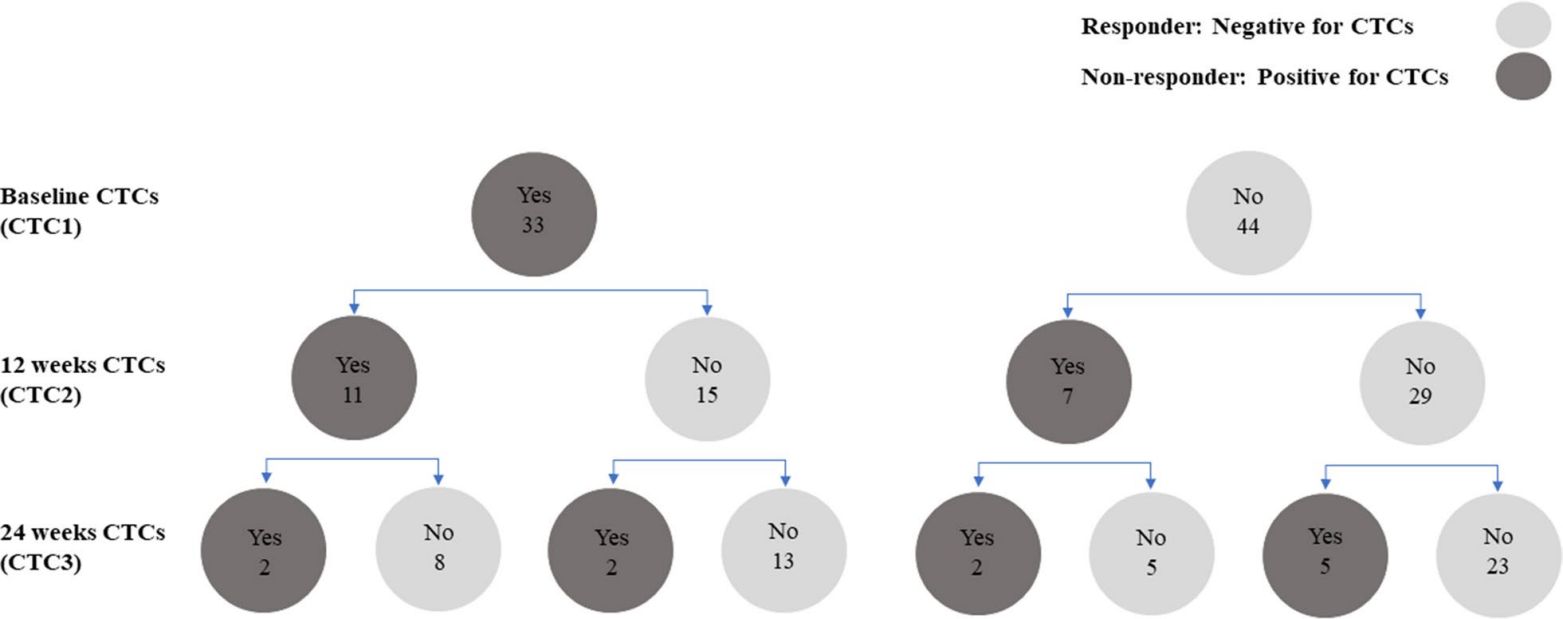
CyCAR criteria based on the status of CTCs along the treatment. Dark grey circles identify persistence of CTCs and are associated with non-responders. Light grey circles identify absence of CTCs and are associated with responders. Numbers in each circle = N

CyCAR results were based on the status of CTCs during the treatment (Fig. 1). Thus, a patient was classified as a responder (favorable response) if he was negative at baseline and continued negative for CTCs at 12 weeks. However, a patient was classified as a non-responder (non-favorable response) if he showed CTCs at 12 weeks. In the same way, the patient was classified as responder if he was negative for CTCs at 24 weeks, but he was classified as non-responder if he showed CTCs at 24 weeks.

### Isolation and enumeration of CTCs

Peripheral blood was collected for CTCs evaluation before the initiation of therapy (baseline) (CTC1) and subsequently at 12 (CTC2), and 24 (CTC3) weeks after initiating the treatment.

For CTCs enrichment and detection, 10 ml of peripheral blood was collected from each mCRC patient and processed according to the protocols based on immunomagnetic selection and established by our group [14].

### Enumeration and characterisation of CTCs by CK and VEGFR expression

Samples containing CTCs^CK+^ were characterized for VEGFR expression by double immunofluorescence (IF) following our standard protocols [14]. We also analyzed 17 healthy blood donors and a colon cancer cell line (HT29) to test the performance of the assays.

### Fluorescence microscopy

**Fig. 2:**
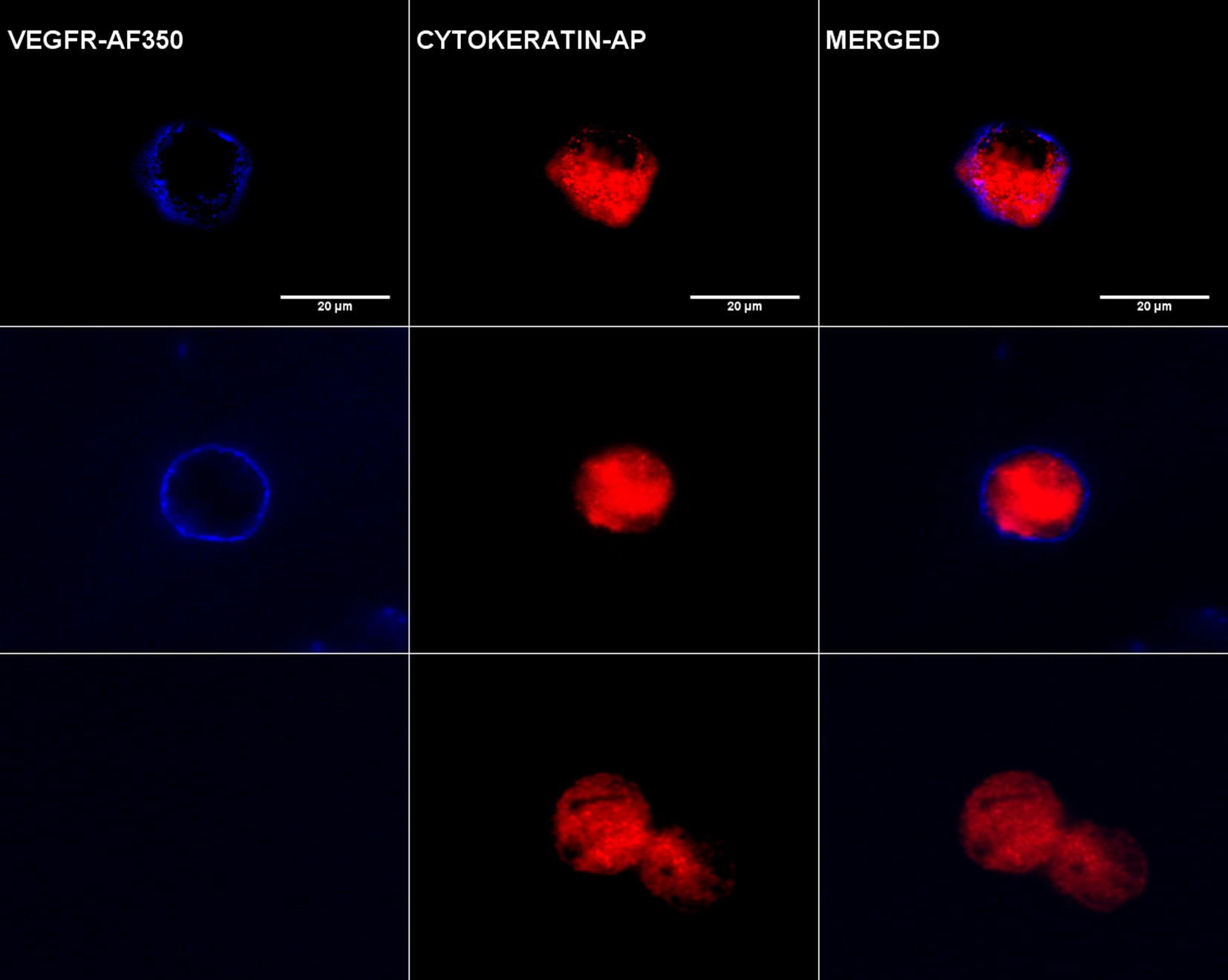
Image gallery after isolation, cytomorphological analysis and detection of cytokeratin-positive tumor cells (CK+, red staining) and vascular endothelial growth factor expression (VEGFR, blue staining). Top row shows HT29 cell tumor line used as a positive control for VEGFR expression. Middle (patient 1) and bottom (patient 2) rows show an example of heterogeneous expression of VEGFR in two different patients: patient 1 shows positive VEGFR expression in a CTC and patient 2 shows negative VEGFR expression in another CTC. VEGFR-specific immunofluorescence (IF) CTCs were determined with Alexa Fluor^®^ 350

Cytospins were previously analyzed for the presence of CTCs under direct light microscope to identify red stained pan-CK cells. Then, samples were observed using a computerized fluorescence microscope Zeiss AXIO Imager. A1 to detect cells with intense VEGFR staining. VEGFR positive cells (CTCs^CK+VEGFR+^) showed an intense blue fluorescence signal on the surface (Fig. 2).

### Statistical analysis

CTCs were assessed as a continuous and a binary variable (presence/absence). The cut-off limit for CTC status as positive was CTCs ≥ 1. The relationships between CTCs and other variables were ascertained using Fisher’s exact test. The Wilcoxon signed-rank test was used to compare CTCs measured at two different times and the Cochran’s Q test to compare presence of CTCs at three different times. The influence of clinicopathological variables on PFS and OS was measured by univariate and multivariate Cox Proportional-Hazards Regression. We applied the criterion of more than a 10% change in the CTC coefficient estimate [15] for the selection of variables to be included in the multivariate model.

## Results

The study was performed in 77 patients with metastatic cancer of colon and rectum (59.7% and 40.3% respectively). The median age was 61 years and 51% of patients were men. Only 12 patients (15.6%) presented synchronous metastases while 65 (84.4%) presented metachronous metastases (metastases developed at least 12 months after the primary tumor) (Table 1). In this way, 59 (76.6%) patients developed liver metastasis, 8 (10.4%) lung metastasis and the remaining 10 (13%) developed metastasis in other organs as bone, lymph nodes or adrenal glands. Nevertheless, no relation was found between levels or status of CTCs and a predisposition to metastasis between organs (p > 0.05).

### Dynamic fluctuation of CTCs during follow-up and correlation with clinicopathological characteristics

CTCs were detected in 33 of 77 (42.8%) patients at CTC1, in 18 of 62 (29%) patients at CTC2 and finally, in 12 of 66 (18.2%) patients at CTC3 (blood samples of some patients were lost due principally to the death of the patients, except for two of them, which could not be analyzed due to sample analysis problems). According to the data, we observed a significant decrease in number of patients with CTCs along the follow-up (CTC1–CTC3) (p = 0.015) and between extraction points CTC1 vs. CTC2 (0.019) and CTC1 vs. CTC3 (0.003). This way, the mean number of CTCs varied from an initial 1.5 cells per 10 ml of peripheral blood [standard deviation (SD): 1.5; range 0–8] at CTC1, 1.7 cells (SD: 8.1; range 0–64) at CTC2, to a reduced number of 0.5 (SD: 1.5; range 0–9) at CTC3 (Table 2).

**Table 2:** Dynamic fluctuation of CTCs in patients according to VEGFR status during follow-up

	CTC1 (baseline)	CTC2 (12 weeks)	CTC3 (24 weeks)	*p* (CTC1 vs CTC2)	*p* (CTC1 vs CTC3)	*p* (CTC1–CTC3)
Patients with CTCs (CTCs^CK+^) N (%)	33 (42.8%)	18 (29%)	12 (18.2%)	0.088	0.005	0.015
Mean number of CTCs^CK+^ (SD; range)	1.5 (1.5; 0–8)	1.7 (8.1; 0–64)	0.5 (1.5; 0–9)	0.019	0.003	
Patients with CTCs^CK+VEGFR+^) N (%)	23 (69.7%)	7 (38.9%)	5 (41.7%)	0.102	0.317	0.368
Mean number of CTCs^CK+VEGFR+^ (SD; range)	1.4 (1.5; 0–5)	0.6 (1.05; 0–3)	0.5 (0.8; 0–2)	0.120	0.317	

CTCs: circulating tumor cells; CK: cytokeratin; VEGFR: vascular endothelial growth factor receptor; SD: standard deviation; *p*: *p* value

Correlation between CTC1 presence, CTC1^VEGFR^ status and clinic-pathological characteristics is summarized in Table 1. The primary tumor resection showed a significant correlation with CTC1 presence (*p *= 0.019). 26 of 49 (53.1%) patients who underwent primary tumor resection were CTC1+, while only 7 of 28 (25%) patients without primary tumor resection were CTC1. Despite finding higher percentage of CTC1+ patients with wild-type K-RAS tumors than patients with mutated K-RAS tumors (54% vs. 31% respectively), there was no significant correlation between them (*p *= 0.061). Lower levels of basal Ca 19.9 significantly correlated with CTC1 presence (*p *= 0.034). Therefore, 22 of 41 (53.7%) patients with standard levels of Ca 19.9 showed CTC1 presence but only 9 of 32 (28.1%) did it in the higher levels group (Table 1).

### Dynamic fluctuation of CTCs according to VEGFR status and correlation with clinic-pathological characteristics

At baseline status, CTC1^CK+VEGFR+^ were detected in 23 of 33 (69.7%) patients. At the second extraction, we found CTC2^CK+VEGFR+^ in 7 of 18 (38.9%) patients, and finally, we found CTC3^CK+VEGFR+^ in 5 of 12 (41.7%) patients. The mean number of CTC1^CK+VEGFR+^ was 1.4 per 10 ml of peripheral blood (SD: 1.5; range 0–5), 0.6 CTC2^CK+VEGFR+^ (SD: 1.05; range 0–3) and 0.5 CTC3^CK+VEGFR+^ (SD: 0.8; range 0–2) (Table 2). During follow-up, although it was not statistically significant, we noted a decrease not only in the percentage of patients with CTC^CK+VEGFR+^, but also in the number of CTC^CK+VEGFR+^ detected per patient (Table 2). However, no significant correlation was found between CTC1^CK+VEGFR+^ status and any clinic-pathological characteristics of the patients, including K-RAS status (Table 1).

**Fig. 3:**
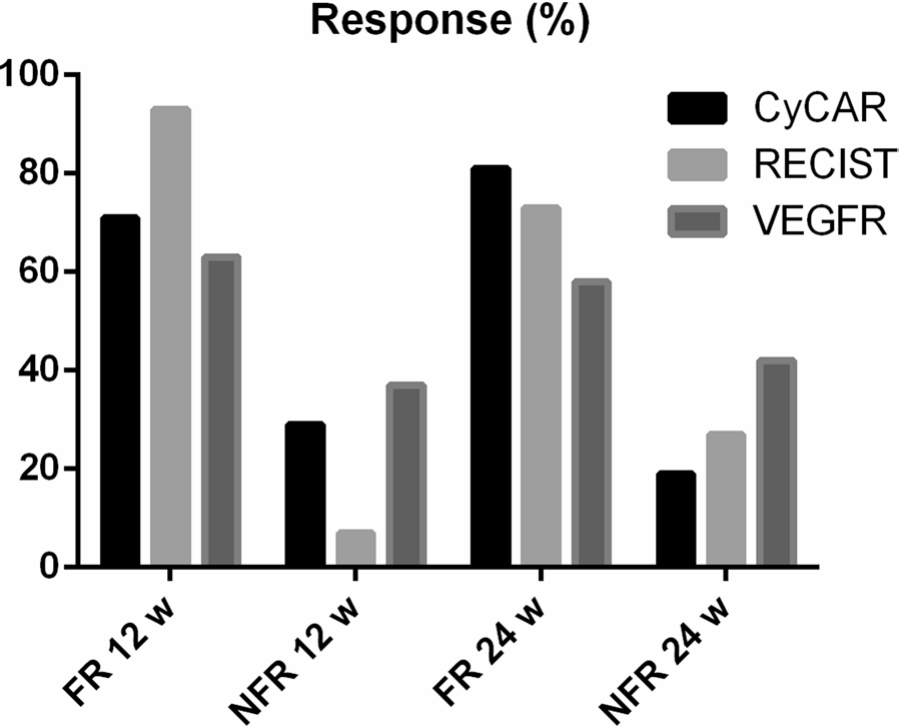
Evaluation of FOLFOX–bevacizumab response by RECIST and CyCAR criteria together with CTCs VEGFR status. *FR* favorable response, *NFR* non-favorable response, *w* week

We compared the previously described CyCAR results to RECIST, in order to evaluate the response at 12 and 24 weeks. We evaluated responses in 62 patients at 12 weeks (Fig. 3). According to RECIST, 4 of the 62 (6.45%) patients showed non-favorable responses, while 58 of the 62 (93.55%) patients showed a favorable response. The CyCAR (CTC2) responses showed similar results, with 18 of the 62 patients (29%) developing non-favorable responses and 44 of the 62 (71%) patients developing a favorable one. As a result, we found no significant differences between the assessment response by RECIST and CyCAR at 12 weeks (*p *> 0.05).

At 24 weeks, we evaluated responses in 65 patients. In this case, according to RECIST, 13 of the 65 (20%) patients showed non-favorable responses, while 52 of them (80%) showed a favorable response. On the other hand, regarding CyCAR evaluation (CTC3), 12 of the 65 patients (18.5%) showed non-favorable responses while 53 of them (81.5%) showed a favorable response. In the same way, we found no significant differences between both assessment criteria (*p *> 0.05).

### Correlation between RECIST and CyCAR based on CTCs VEGFR status to evaluate response to FOLFOX–bevacizumab

CyCAR results based on VEGFR status in CTC2 and CTC3 was compared to RECIST evaluation at 12 and 24 weeks. At the first response evaluation time point (12 weeks), 18 patients were analyzed, while at the second response re-evaluation (24 weeks), only 12 of the 18 patients remained CTC3+ and were analyzed (Table 2).

According to RECIST at 12 weeks, 17 of the 18 (94.4%) patients showed favorable responses and only 1 of these 18 patients (5.6%) developed a non-favorable response. Using CyCAR, 7 of the 18 CTC2^CK+^ patients were VEGFR+, with 6 of the 7 (85.71%) patients with CTC2^CK+VEGFR+^ showing favorable responses, and just 1 of the 7 (14.29%) patients showing a non-favorable response. The remaining 11 CTC2^CK+VEGFR−^ patients were identified as responders (*p *> 0.05) (Fig. 3).

On the other hand, according to RECIST, at 24 weeks 9 of the 12 (75%) CTC3+ patients showed favorable responses with only 3 of them (25%) developing a non-favorable response. According to CyCAR, 5 of the 12 (42%) patients were CTC3^CK+VEGFR+^ and all of them showed a favorable response. However, in the CTC3^CK+VEGFR−^ group (7/12), 4 (57%) were identified as responders and three as non-responders (43%), showing no statistical differences between the groups (*p *> 0.05).

### Prognostic significance of CTCs detection

36 of the total 74 patients enrolled in this study died from disease progression. Median overall survival for these 36 patients was 18.3 months, compared to 37.5 months for the remaining 38 patients.

In the univariate analysis of the factors associated with OS (Table 3), we did not find any positive association between RECIST response at 12 weeks and risk of death (HR: 0.26, 95% CI 0.06–1.14; *p *> 0.05). In the same way, there was no correlation between CyCAR at 12 weeks (CTC2) and risk of death (HR: 1.36; 95% CI 0.62–3.01; *p *> 0.05).

**Table 3:** Univariate and multivariate Cox regression analysis for overall survival (OS)

Characteristics	Univariate analysis				Multivariate analysis		
	Median OS (months)	HR	95% CI	*p*	HR	95% CI	*p*
All patients	30.3						
Age							
< 55	22.4	1.72	0.88–3.37	0.113			
≥ 55	23.4						
Gender							
Male	23.3	0.70	0.36–1.38	0.307			
Female	20						
Primary tumor location							
Colon	22.4	0.78	0.39–1.58	0.497			
Rectum	22.85						
Primary tumor surgery							
Yes	39.4	0.17	0.08–0.37	< 0.0001	0.37	0.12–1.11	0.075
No	11.6						
Response (12 weeks) (RECIST)							
Favorable	41.6	0.26	0.06–1.14	0.074	0.1	0.02–0.58	0.011*
Adverse	16.8						
Response (24 weeks) (RECIST)							
Favorable	48.6	0.51	0.22–1.20	0.123			
Adverse	14.8						
K-RAS status							
Mutated	36.9	1.95	0.96–3.97	0.064	1.58	0.68–3.68	0.289
Wild-type	55.4						
Metastasis surgery							
Operated	68.4	0.35	0.15–0.81	0.014	0.39	0.12–1.24	0.111
Non-operated	31.1						
Synchronous metastasis							
Yes	32.1	15.89	2.04–123.35	0.008	18.33	1.52–221.18	0.022*
No	68.4						
CEA Basal							
High	39.4	1.96	0.84–4.57	0.121			
Standard	55.4						
Basal Ca 19.9							
High	22.4	1.68	0.87–3.24	0.123			
Standard	55.4						
CTC1							
Favorable	21.6	0.32	0.72–2.79	0.319			
Adverse	23.5						
CTC1 VEGFR							
Yes	66.5	0.53	0.25–1.15	0.109			
No	36.9						
CTC2							
Favorable	39	1.36	0.62–3.01	0.442			
Adverse	55.4						
CTC2 VEGFR							
Yes	36.9	1.44	0.31–6.66	0.640			
No	41.6						
CTC3							
Favorable	55.4	0.52	0.22–1.26	0.149	0.35	0.12–0.99	0.049*
Adverse	22.4						
CTC3 VEGFR							
Yes	22.4	1.04	0.23–4.81	0.958			
No	30.9						

*: Statistically significant

CTC: circulating tumor cell; OS: overall survival; HR: hazard risk; CI: confidence interval; VEGFR: vascular endothelial growth factor receptor positive; RECIST: Response Evaluation Criteria in Solid Tumors; *p*: *p* value

At 24 weeks, we found no positive correlation between the risk of death and CyCAR and RECIST criteria (HR: 0.221; 95% CI 0.22–1.12; *p *> 0.05 and HR: 0.5; 95% CI 0.22–1.26; *p *> 0.05, respectively).

Then, we analyzed the OS in responder and non-responder patients by RECIST and CyCAR. We observed that the median OS in responder vs. non-responder patients was 48.6 vs. 14.8 months according to RECIST and 55.4 vs. 22.4 months by CyCAR, evaluated at 24 weeks. In conjunction with these results, the presence of CTCs after treatment identified those patients with worse OS, which was in concordance with the results obtained by RECIST.

In the univariate analyses, metastasis surgery was significantly associated with a higher overall survival (HR = 0.35; 95% CI 0.15–0.81; *p *= 0.014). In the same way, we found that the presence of synchronous metastasis decreased the OS when compared to the presence of metachronous metastasis (HR = 15.89; 95% CI 2.04–123.35; *p *= 0.008).

The multivariate analysis included the significant risk factors from the univariate analysis. Response assessment at 12 weeks by RECIST, response assessment at 24 weeks by CyCAR, and synchronous metastasis variables were independent prognostic factors associated with OS (Table 3). According to the PFS, RECIST response at 12 weeks was the only significantly associated variable in the multivariate analysis.

## Discussion

RECIST has been adopted as the standard method for tumor assessment and helps in clinical decision making. Deciding and then monitoring the effectiveness of individual therapies in mCRC patients is currently very challenging, as a result of the high prevalence of lymph abdominal, peritoneal, serous and pleural metastasis, which are particularly difficult to evaluate by RECIST [16]. In addition, the thresholds (of response or progression) for predicting differences in survival in treated patients probably differ according to the type of treatment and the type of cancer [17]. For example, targeted molecules such as anti-VEGF or anti-EGFR often induce only small size changes, whereas patient survival is significantly prolonged. Therefore, specific criteria for certain diseases or treatments are necessary, as the current biomarkers and imaging evaluation options to monitor and register treatment clinical responses do not yet allow for optimal management of individual patients yet [18].

The hypothesis that CTCs are a fundamental prerequisite to metastasis was first proposed in the mid-nineteenth century (1869) by Thomas Ashworth, an Australian pathologist [19]. The characterization of CTCs, derived from a ‘simple’ blood test, have the potential to serve as ‘real-time tumor biopsies’ permitting accurate, up-to-date pictures of tumor activity, without the need for invasive tissue biopsies. Furthermore, CTCs can be analyzed on a serial basis, allowing real-time identification of emerging treatment ‘resistance profiles’, and consequently, being of significant assistance to the radiological assessment of tumor responses [20].

We performed this study in 77 patients with metastatic colorectal cancer treated in a homogeneous manner. The overall response rate to treatment was 24 months and median overall survival was 23.3 months, which did not differ from that expected [21]. In our study, we cover responses of metastatic colon cancer patients to bevacizumab, assessing and comparing CTCs and standard evaluation criteria, and we found comparable results to other published studies.

In our study, we compared CyCAR to RECIST in the same mCRC patient cohort, to determine the specific importance of CyCAR. In addition, we analyzed the association between OS and PFS with different clinic-pathological characteristics such as the presence of CTCs, at follow-up times.

The correlation between K-RAS tumor status and the presence of CTCs was analyzed, finding borderline significant association (*p *= 0.06) between higher percentages of CTC positive patients with K-RAS wild-type tumors. Similar results were reported in a recent study with 24 metastatic colon cancer patients by Das et al. [22]. In the same way, Buim et al. [23] observed that only 9 of their 23 CTCs positive patients had K-RAS mutations in their corresponding primary tumors. Our results, together with the aforementioned studies, suggest that the dissemination of CTCs is an independent process to K-RAS status in the primary tumor.

On the other hand, we detected a direct association between the presence of CTCs and primary tumor surgery. In fact, most patients undergoing primary tumor surgical treatment were positive for the presence of CTCs. These results concur with several studies, which demonstrated that primary tumor resection can stimulate cellular proliferation of residual colorectal tumors [24]. However, other studies demonstrated that primary tumor resection improves OS in metastatic colon patients [25]. Interestingly, the absence of CTCs in mCRC has been also associated with a higher OS in several studies [26].

Additionally, besides analyzing the presence of CTCs, we also analyzed their heterogeneous VEGFR expression. VEGF is the target of bevacizumab, a humanized anti-VEGF monoclonal antibody (Bevacizumab; Avastin). This drug has been approved by the FDA in the first and second-line colorectal cancer setting, in combination with chemotherapy. Bevacizumab acts by selectively binding circulating VEGF, inhibiting the binding of VEGF to its cell surface receptor, and in this way preventing its activation. Despite this immobilization of VEGF, we found no association between VEGFR status in CTCs with the response to treatment, even though we describe a no statistically significant reduction in the cell subpopulations of CTC^CK+VEGFR+^ and a decrease in the number of CTC^CK+VEGFR+^ patients, before and after treatment. These results suggest that the treatment could be effective at removing VEGFR+ tumor cell populations, but also selecting VEGFR− populations. Interestingly, Simiantonaki et al. [27] demonstrated that negative VEGFR-1 expression was significantly associated with lymphogenous and haematogenous metastases. In the same way, Hanrahan et al. [28] found a significant increase of VEGFR-1 mRNAs in T3/T4 colorectal carcinomas compared to lymphogenously metastasising tumors, and Lebok et al. [29] showed that low VEGFR-1 tumor expression was associated with lower survival and correlated with an advanced disease status in breast cancer. According to these results, we expected CTCs^CK+VEGFR−^ patients to have a worse prognosis; however, we did not observe any difference in OS or PFS between patients with CTCs^CK+VEGFR+^ and CTCs^CK+VEGFR−^. In this context, Senger et al. [30] demonstrated the existence of autocrine VEGF signaling in human tumors that might reflect the importance of VEGF for sustaining the self-sufficiency or autonomy of tumor cells, especially relevant to aggressive cancers and to the biology of cancer stem cells. Autocrine VEGF signaling is also closely associated with tumor dedifferentiation and with epithelial–mesenchymal transition (EMT), which are processes involved in the genesis of cancer stem cells. In this context, it has been suggested that autocrine signaling blocking by bevacizumab is modest and that its action can be improved by combining autocrine signaling blocking therapies [31]. These mentioned analyses, could explain why we cannot find differences in the OS and PFS between positive and negative VEGFR expression in CTCs.

The principal objective of this study was to compare CyCAR with RECIST to predict bevacizumab response. In our analysis, we observed no differences between both criteria. In fact, we detected similar results when we compared the CyCAR and the RECIST response at 12 weeks. We also obtained similar results when analyzing the assessment response at 12 (71% vs. 93%) and 24 weeks (82% vs. 80%) for CyCAR and RECIST respectively.

Our results suggest that the presence of CTCs at 12 and 24 weeks can be useful predictive markers, and used as a complementary tool with RECIST, even in those patients where RECIST can be more difficult to apply.

We also analyzed the predictive role of the presence of CTC^CK+VEGFR+^ to assess the treatment response in these patients. However, due to the low number of CTC^CK+VEGFR+^ patients we have not been able to demonstrate that the presence of VEGFR in CTCs is a predictive factor of tumor response to FOLFOX–bevacizumab. However, it would be important to note that one the principal limitation of this study is the methodology used to isolate these CTCs, since this isolation is based on the epithelial markers expression, therefore, we could be losing an important subpopulation of CTCs, which are under EMT process. Recently, Zhang et al. [32], demonstrated that VEGF expression rate in mesenchymal CTCs was significantly higher than that of epithelial CTCs, which suggested that VEGF may be correlated with tumor malignancy “and probably with the resistance”.

Although these results suggest the value and importance of CTCs for monitoring treatments, further studies are necessary including, not only more patients, but also a deeper study about the autocrine and paracrine activity involved in the activation of VEGF and its role in the migration and proliferation of circulating tumor cells.

## Conclusion

These results suggest that CyCAR is similar to RECIST criteria at evaluating the response in metastatic colorectal carcinoma. Although RECIST is useful for evaluating treatment efficacy in clinical trials and practice, it has some limitations. This way, we propose the use of CyCAR and RECIST combination for a better response prediction of metastatic colorectal carcinoma, in special when RECIST is limited.

## Abbreviations

CI: confidence interval
CRC: colorectal cancer
CTC1: baseline CTCs
CTC2: CTCs at 12 weeks after treatment initiation
CTC3: CTCs at 24 weeks after treatment initiation
CTCs: circulating tumor cells
CyCAR: Cytologic Criteria Assessing Response
HR: hazard ratio
IF: immunofluorescence
mCRC: metastatic colorectal cancer
OS: overall survival
PFS: progression-free survival
RECIST: Response Evaluation Criteria in Solid Tumors
SD: standard deviation
VEGF-A: vascular endothelial growth factor A
